# Supplementary material for: Cdc42 mobility and membrane flows regulate fission yeast cell shape and survival
Source: Nat Commun. 2024 Sep 27;15:8363. doi: 10.1038/s41467-024-52655-1 (PMC11437197; doi:10.1038/s41467-024-52655-1)
Supplement: Supplementary file 1 — Supplementary information [file 41467_2024_52655_MOESM1_ESM.pdf]

Supporting Information for

Cdc42 mobility and membrane flows regulate fission yeast cell shape and survival

David M. Rutkowski[1], Vincent Vincenzetti[2], Dimitrios Vavylonis[1, *] and Sophie G. Martin[2, 3, *]

* Co-corresponding authors: Sophie G Martin, Dimitrios Vavylonis
Email: sophie.martin@unige.ch; vavylonis@lehigh.edu

This PDF file includes:

Figures S1 to S10
Tables S1 to S4
SI References

Other supporting materials for this manuscript include the following:

Movies S1 to S4

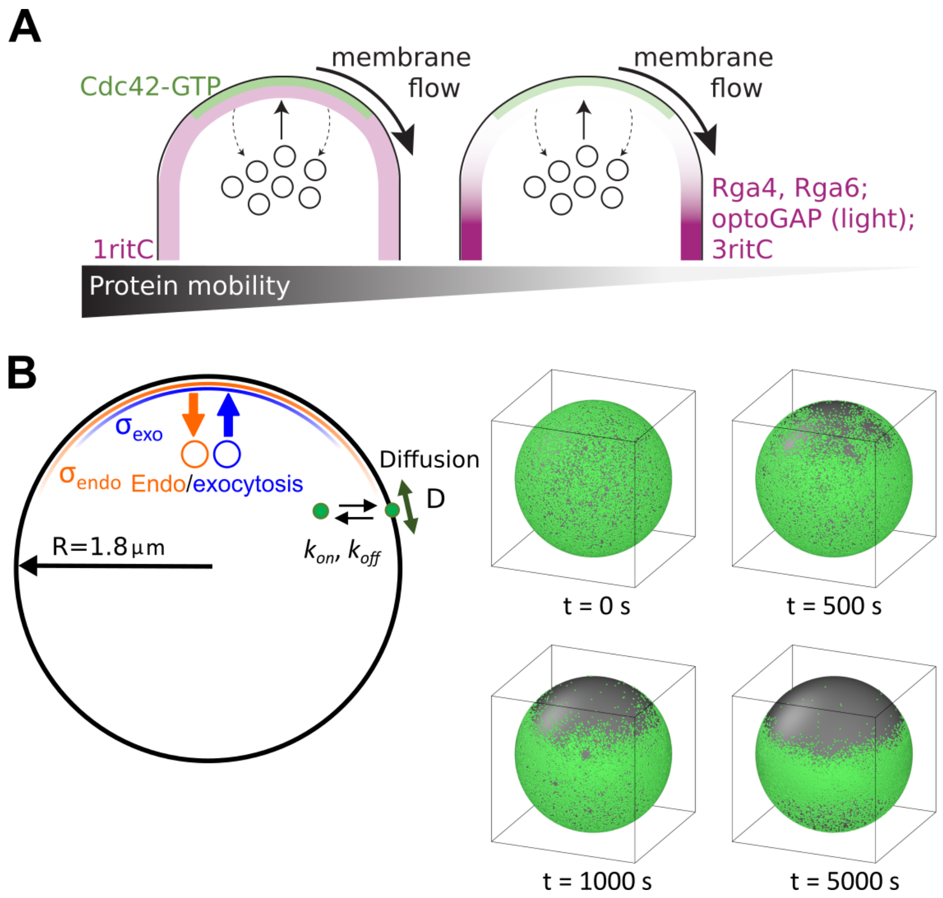

Figure S1. In plane membrane flow due to localized secretion and broader endocytosis can lead to peripheral membrane protein depletion.

A. Schematics showing that high mobility proteins (Cdc42-GTP, 1ritC) are able to keep up with membrane flows due to exocytosis and endocytosis and remain at the tip of growing *S. pombe* cells. Low mobility proteins (Rga4/6, optoGAP which dimerizes under light, and 3ritC) are displaced by the membrane flow. Modified from [1] ©The Authors, some rights reserved; exclusive licensee AAAS. Distributed under a Creative Commons Attribution NonCommercial License 4.0 (CC BY-NC) http://creativecommons.org/licenses/by-nc/4.0/ **B.** Computational model of membrane flow, reproduced from [1], simulated as stochastic, area-conserving exocytosis and endocytosis events centered at a point of spherical domain, with inert particles undergoing diffusion and binding/unbinding. Model shows depletion of initially uniform particle distribution away from the region where exocytosis and endocytosis are occurring, for sufficiently small diffusion coefficients and unbinding rates. The depletion phenomenon occurs when the exocytosis region is narrower than the endocytosis even when the overall rates of membrane secretion and internalization are equal to each other. From [1] ©The Authors, some rights reserved; exclusive licensee AAAS. Distributed under a Creative Commons Attribution NonCommercial License 4.0 (CC BY-NC) http://creativecommons.org/licenses/by-nc/4.0/.

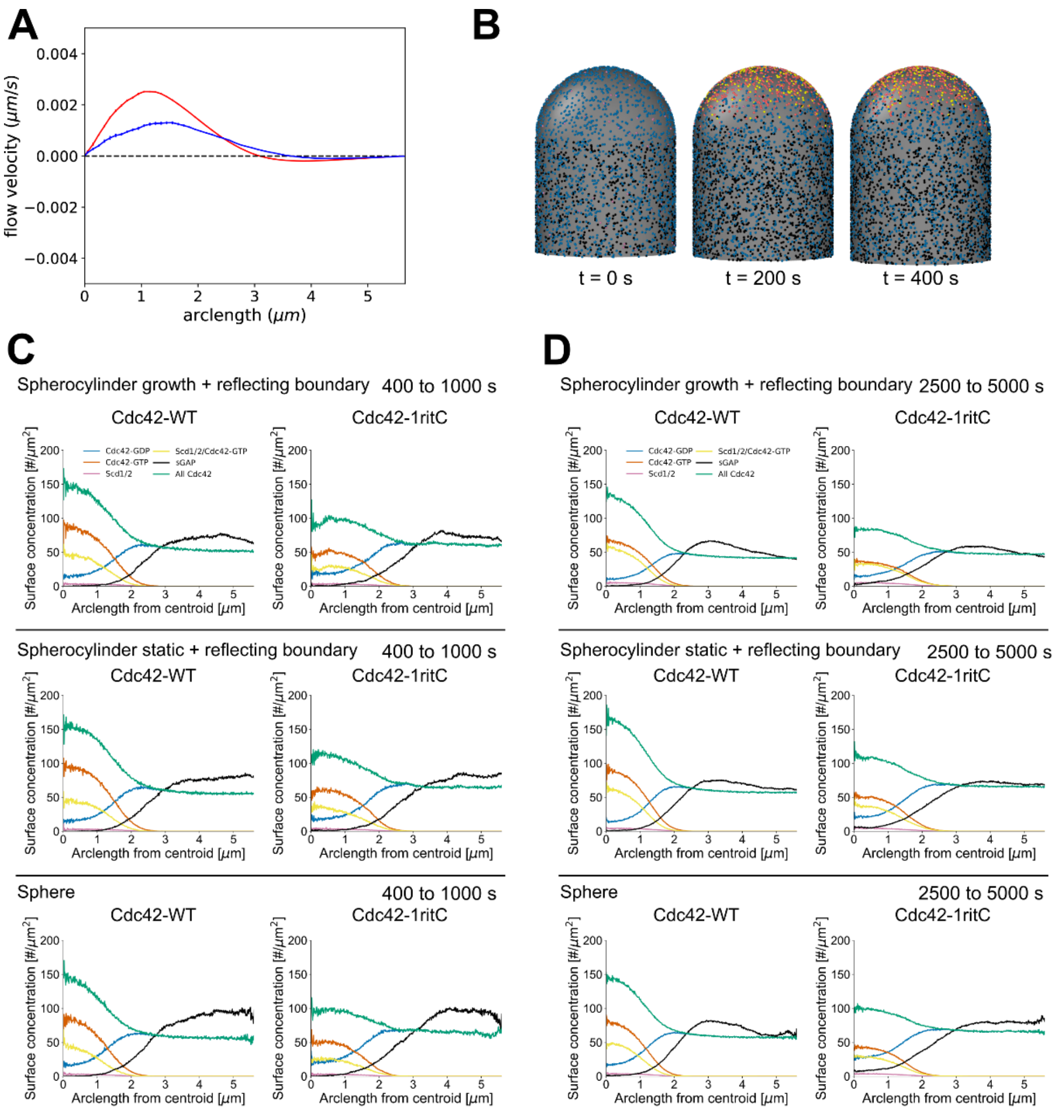

Figure S2. Patch formation at the pole of spherocylinder domain.

A. Average membrane flow velocity away from the north pole. This was determined under growth conditions from random exo/endocytosis events driven by Cdc42-GTP. In these calculations, the width of Cdc42-GTP distribution was equal to the average found in simulations of WT Cdc42 (red) and Cdc42-1ritC (blue) on the sphere surface (Figures 1E and 3A). Error bars represent standard deviation. **B.** Spherocylinder growth simulations where the cylinder surface terminates in a reflecting boundary (WT case). The shifting downwards of this boundary is associated with net cell growth (net elongation rate of 0.031 μm/min). All sGAP particles are initiated in the cylinder portion of the surface to avoid transient patch formation outside the tip region. **C-D.** Concentration profiles at intermediate times (400 to 1000 s; C) and longer times (2500 to 5000 s; D) after patch formation, comparing polarization under WT and 1ritC conditions for a growing spherocylinder, a static spherocylinder, and a sphere, using particle displacements due to endocytosis and exocytosis as in panel (A). A peak of sGAP at ~ 3-3.5 μm is observed at long times due to flow displacement. The Cdc42 patch weakens in the growing spherocylinder due to growth-induced particle dilution. Source data provided as Source Data file.

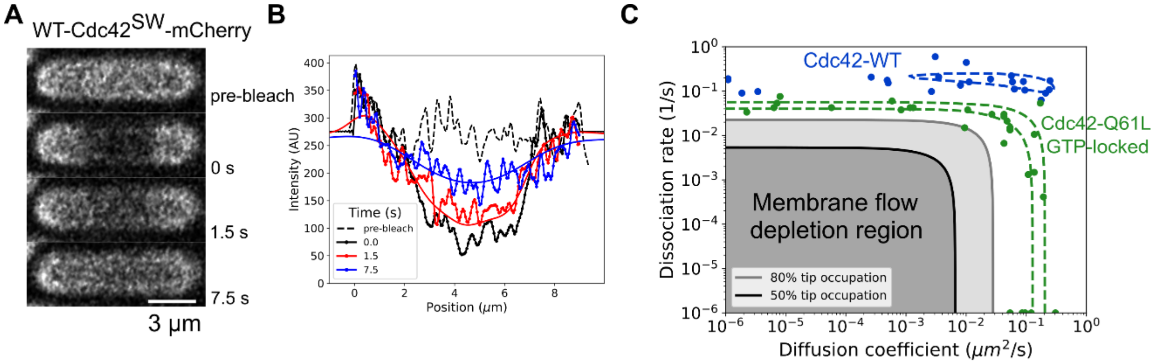

Figure S3. FRAP fits to extract diffusion coefficients and membrane dissociation rates.

A. Photobleaching of a rectangular region along the cell sides. Confocal section at the cell surface for WT Cdc42-mCherry^{SW}. Similar FRAP experiments were performed for cells in Figure 2B. **B.** Recovery traces over time and fit (solid lines) for cell in panel (A). The fitted intensity was measured by projecting the intensity within a box of cell width size perpendicularly to the long axis of the cell. **C.** Best fit parameters for membrane unbinding and diffusion from side FRAPs of WT Cdc42 (as in Figure 2B) and GTP-locked Cdc42^{Q61L}-mCherry^{SW}. Each point corresponds to individual cell best fit. Dashed line regions are drawn based on an average across all cell recoveries (R^2 measure = 96% of the maximum, see *Materials and Methods*). The values corresponding to the maximum averaged R^2 are shown as model parameter values for Cc42-GDP and Cdc42-GTP in Table S2. WT Cdc42 (primarily consisting of Cdc42 in its GDP form) diffusion and dissociation constants are comparable to ~0.2 μm^2/s and ~0.03 /s measured using an alternative FRAP method [2]. Cdc42^{Q61L} fits indicate a reduction of Cdc42-GTP dissociation constant by an order of magnitude or more compared to Cdc42-GDP. Prior half-tip Cdc42 FRAP experiments indicated a reduction in Cdc42-GTP diffusion coefficient at cell tips [2]; we could not detect (or exclude) such a reduction with the current method due to the large scatter of data along the *D* axis. Source data provided as Source Data file.

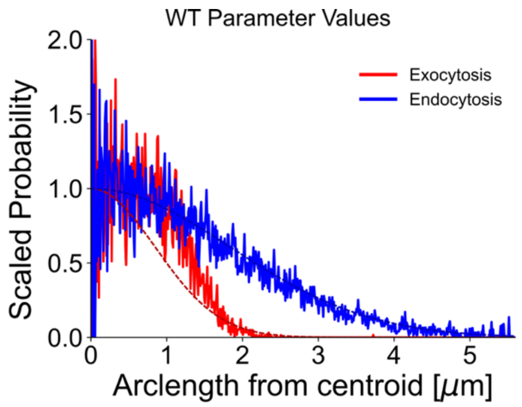

Figure S4. Distribution of exocytosis and endocytosis.

Steady state distribution of exocytosis and endocytosis in WT simulations of Figure 1E (solid lines) versus the experimentally measured distribution (dashed lines) in [1]. The average was calculated similarly to concentration profiles over 5000 s at steady state. Source data provided as Source Data file.

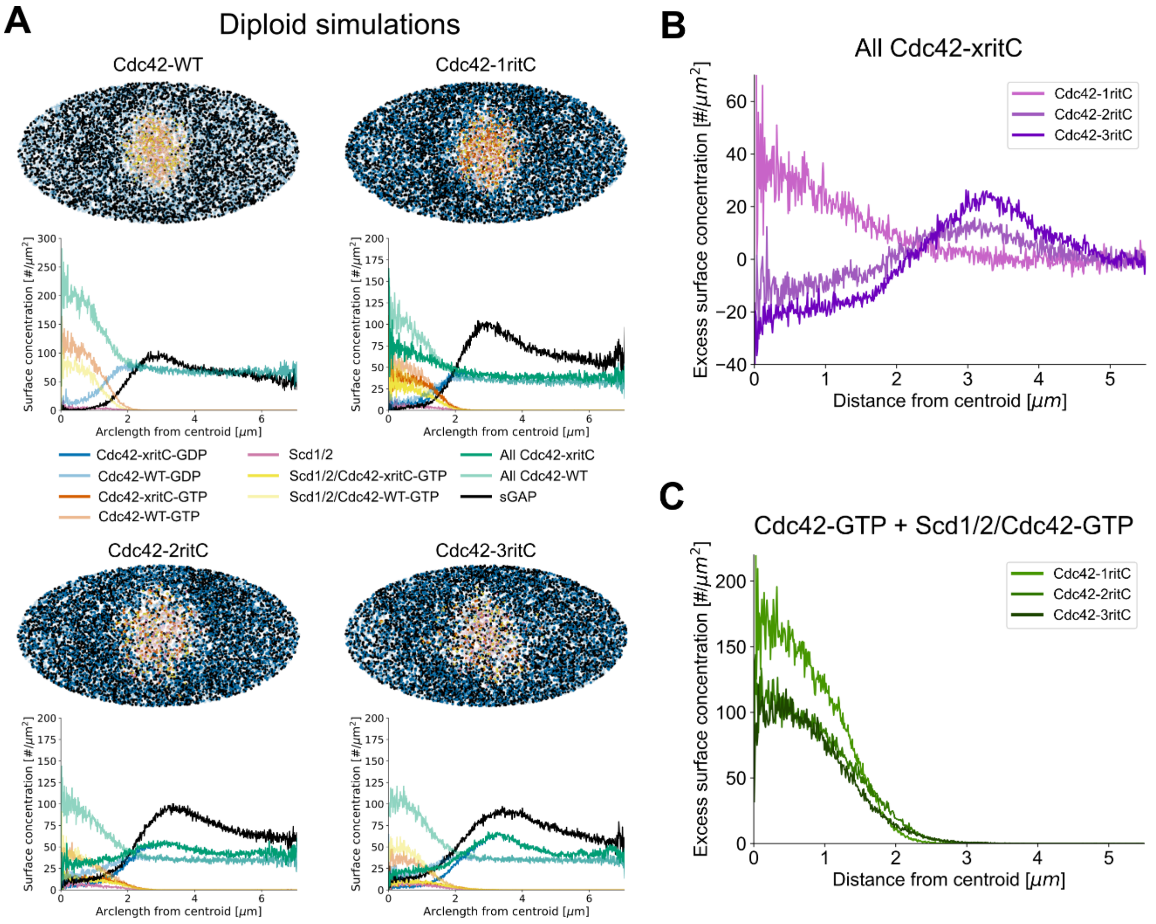

Figure S5. Simulated polarization for diploid cell parameters.

A. Steady state snapshots and concentration profiles from diploid simulations after steady state for WT or where one copy (half of Cdc42) has lower mobility corresponding to either Cdc42-1ritC, Cdc42-2ritC, or Cdc42-3ritC. WT Cdc42 associated particles shown in faded colors. Profiles are averaged over time as in Figure 1. Snapshots were taken after 2000 s. **B.** Side normalized Cdc42 concentration of only the mutant Cdc42 component. **C.** Side normalized Cdc42-GTP concentration for both mutant and WT components. Source data provided as Source Data file.

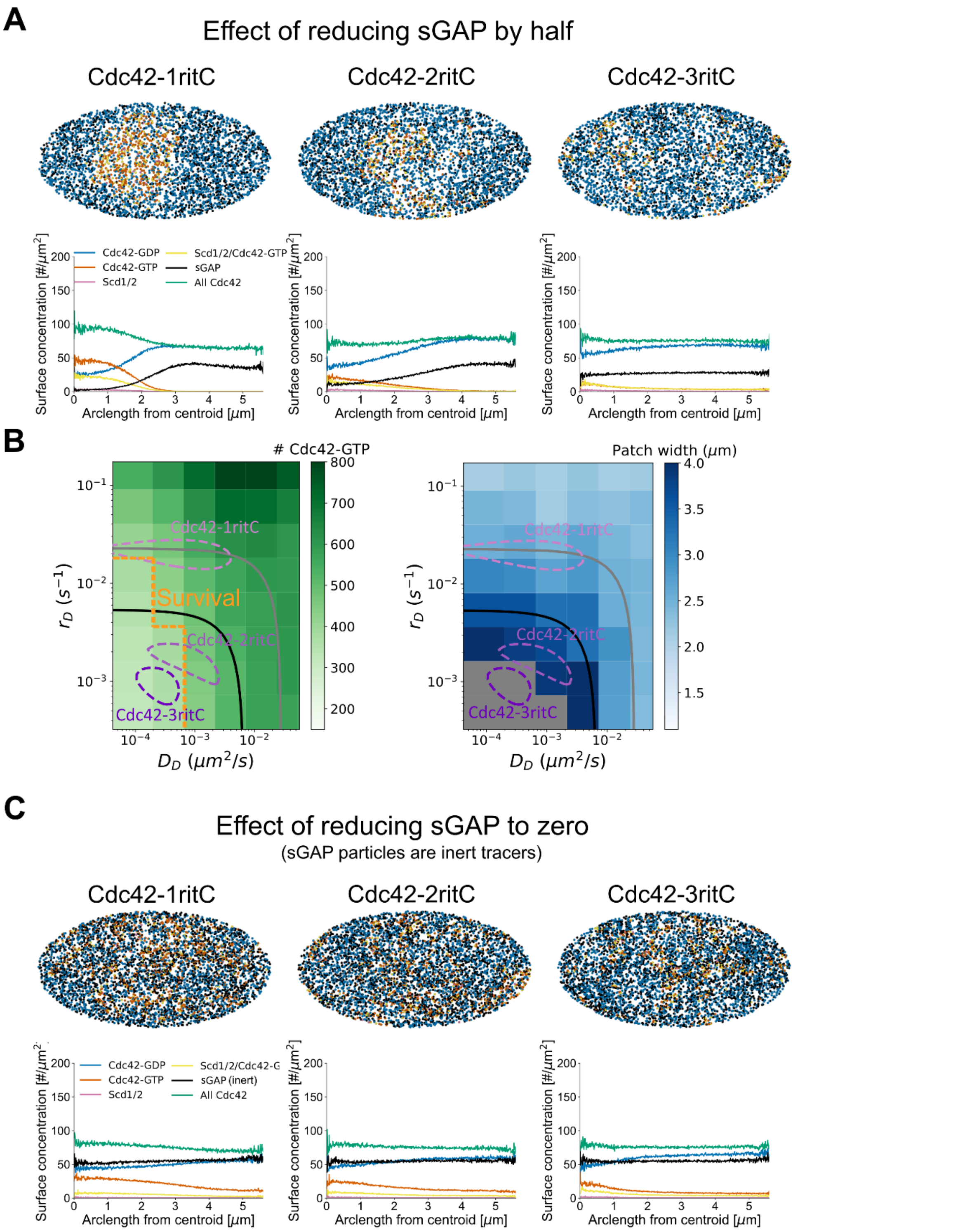

Figure S6. Simulations with reduction of sGAP particle numbers.

A. Steady state snapshots and concentration profiles for simulations where the number of sGAP has been reduced by ½ compared to Figure 1E. The simulation with Cdc42-3ritC shows small transient Cdc42-GTP clusters that are picked up in the concentration profile. However, these small clusters are mobile over the simulation time and do not lead to a depletion of sGAP around

them. Snapshots were taken after 14000 s. **B.** Parameter scan for amount of Cdc42-GTP and the patch width as in Figure 3E, for ½ sGAP simulations. The survival threshold line moves to lower diffusion and detachment rates compared to Figure 3E. **C.** Steady state snapshots and concentration profiles for simulations where the number of sGAP has been reduced to 0. In these simulations passive tracer sGAP particles were added to detect stable polarization. All cases show transient Cdc42-GTP clusters that are picked up in the concentration profile. These small clusters are mobile over the simulation time and do not lead to a depletion of the inert tracer sGAP around them. Snapshots were taken at 15000 s. Source data provided as Source Data file.

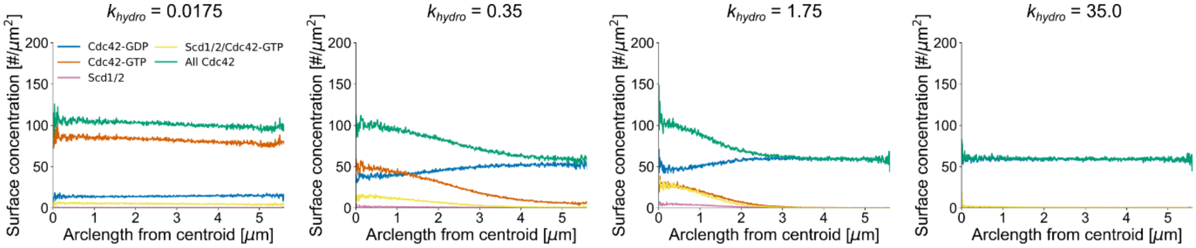

Figure S7. Simulations without sGAP for WT parameters with different values of k_{hydro}.

Without the presence of sGAP, increasing k_{hydro} by around an order of magnitude compared to the reference value (0.175 s^{-1}, Figure 1F) recovers a more focused patch. Increasing or decreasing k_{hydro} too far results in loss of Cdc42-GTP polarization. Source data provided as Source Data file.

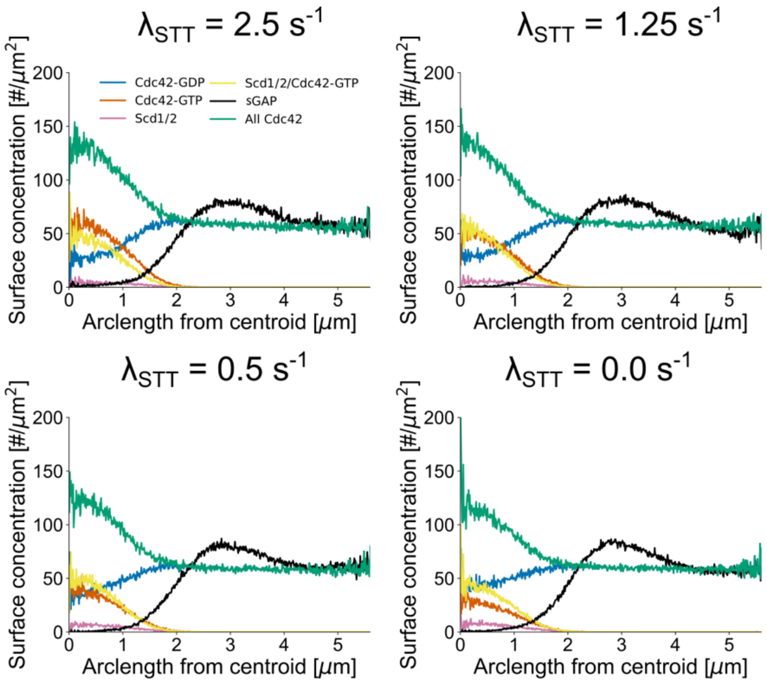

Figure S8. Simulations without non-linear positive feedback form a Cdc42-GTP patch.

Concentration profiles for WT case while reducing non-linear positive feedback. Reducing the non-linear positive feedback term, λ_{STT}, from the reference value of 5 s^{-1} (Figure 1E) decreases the strength of the patch but does not abolish it. Source data provided as Source Data file.

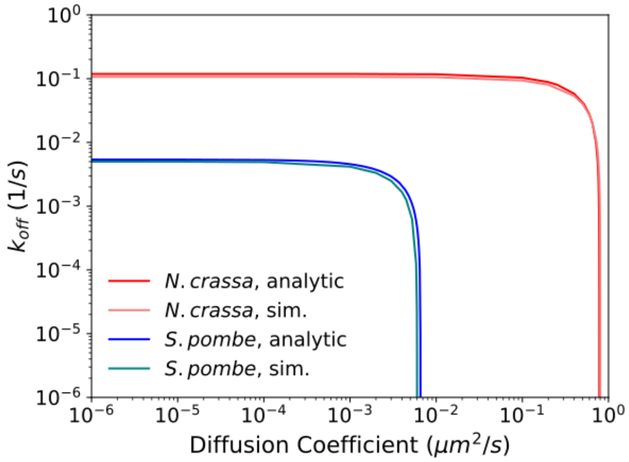

Figure S9. Depletion boundaries for different organisms.

Depletion boundaries (50 % tip occupation as in Figure 1D) for *S. pombe* and *N. crassa* determined by either simulation (lighter lines) or the analytical expression $f_1 D/v < L < f_2 v/k_{off}$ (darker lines), where f_1 and f_2 are numerical prefactors of order unity. Simulation results used passive particles displaced by fixed endocytosis and exocytosis distributions around a fixed point on a sphere as in [1]. For the *N. crassa* simulations we used a sphere with radius 5 µm where exocytosis and endocytosis profile standard deviations were multiplied by 2.78 that for *S. pombe*. Exocytosis and endocytosis rates r_{exo} and r_{endo} were multiplied by 150 and 27 that for *S. pombe*, respectively, in order to match the total rate of membrane addition/removal given in [3]. Vesicle size was assumed to be the same as in Table S3. The values of *α* and *γ* were both set to 1.0. The exo/endocytosis cutoff R_{cutoff} was increased to 5 µm for the *N. crassa* simulations. The analytical expression relates the approximate diffusion coefficient, D, and unbinding rate, k_{off}, needed to see depletion at a given length scale *L* and under a flow rate of v. The values $f_1 = 1$ and $f_2 = 0.333$ lead to an excellent fit between the analytic and passive particle simulation results for the 50% boundary using $L = 2\ \mu m$ and $v = 3 \times 10^{-3}\ \mu m/s$ (corresponding to *S. pombe* values [1]). To connect these two limiting values we assume a form for $D = (1 - \tau k_{off})/(\tau C)$ where τ and C are fixed by the limiting values. For *N. crassa* we set $L = 5\ \mu m$ and determine $v = A_{exo} r_{exo}/(4\pi s) = 0.1604\ \mu m/s$ as the flow rate due to exocytosis at an average exocytic arclength *s* = 1.589 μm based on trafficking measurements [3]. For *N. crassa*, $D < 0.802\ \mu m^2/s$ or $k_{off} < 0.107\ s^{-1}$ leads to reduction of tip occupancy by more than 50%. While the depletion boundary shifts to larger D and k_{off} values for *N. crassa*, this analysis shows that a dissociation rate similar to that of Cdc42-GDP ($0.17\ s^{-1}$) would provide sufficient mobility to withstand the membrane flow. Source data provided as Source Data file.

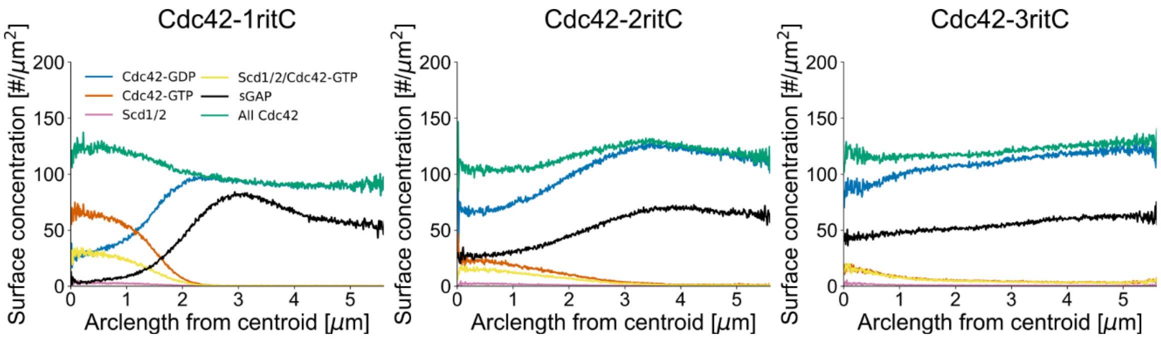

Figure S10. Simulations of Cdc42-xritC constructs as in Figure 3A but with fixed Cdc42-GDP association rate constant, k_D

Instead of using the reference value for k_D given in Table 2 which keeps ~3/5 of the Cdc42 on the plasma membrane, we investigate the effect of keeping k_D fixed at a value of 0.1 s^{-1}. Compared to the profiles in Figure 3A the overall concentration levels are slightly increased, but the result that decreasing Cdc42 mobility leads to weaker patches still holds. Source data provided as Source Data file.

Table S1. Table of model particle reactions (on membrane unless indicated).

Reaction	Associated rate constants
Cdc42-GTP → Cdc42-GDP	k_{hydro}
Cdc42-GTP → Cdc42-GDP (cyto)	r_T
Cdc42-GDP ⇌ Cdc42-GDP (cyto)	k_D, r_D
Scd1/2 + Cdc42-GDP→ Scd1/2 + Cdc42-GTP	λ_{SD}
Scd1/2/Cdc42-GTP + Cdc42-GDP → Scd1/2/Cdc42-GTP + Cdc42-GTP	λ_{STT}
Scd1/2 + Cdc42-GTP ⇌ Scd1/2/Cdc42-GTP	λ_{ST}, r_{ST}
Scd1/2 ⇌ Scd1/2 (cyto)	k_S, r_S
Scd1/2 (cyto) + Cdc42-GTP → Scd1/2/Cdc42-GTP	k_{ST}
Cdc42-GTP + sGAP → Cdc42-GDP + sGAP	λ_{sGAP}

Table S2. Model parameter values for particle reactions.

Variable	Value	Description
Δt	0.001 s	Timestep for simulations
D_T	0.1 μm^2/s	Diffusion coefficient of WT Cdc42-GTP (Cdc42^{Q61L} side FRAP, this work)
D_D	0.1 μm^2/s	Diffusion coefficient of WT Cdc42-GDP (side FRAP, this work)
D_S	0.0025 μm^2/s	Diffusion coefficient of Scd1/Scd2 complex (WT cells) (same as Bem1 [4])
D_{ST}	0.0025 μm^2/s	Diffusion coefficient of Scd1/Scd2/Cdc42-GTP complex (WT cells, same as Scd1/Scd2 complex)
D_{sGAP}	0.001 μm^2/s	Diffusion coefficient of sGAP (assumed to be inside membrane flow depletion region)
r_T	0.015 /s	Membrane dissociation rate of WT Cdc42-GTP (Cdc42^{Q61L} side FRAP, this work)
r_D	0.17 /s	Membrane dissociation rate of WT Cdc42-GDP (side FRAP, this work)
r_S	0.5 /s	Membrane dissociation rate of Scd1/2 (decreased from Bem1 value [4])
r_{ST}	0.5 /s	Membrane dissociation rate of Scd1/2/Cdc42-GTP (decreased from [4])
r_{sGAP}	0 /s	Membrane dissociation rate of sGAP (assumed to be inside membrane flow depletion region)
k_{hydro}	0.175 /s	Background hydrolysis rate (halved from [4] to account for sGAP contribution)
k_D	$\beta(r_T + 2r_D)$	Membrane binding rate of Cdc42-GDP (empirical estimate giving a significant fraction (~3/5) of all WT Cdc42 on plasma membrane at steady state) where $\beta = 0.513$.
k_s	0.01 /s	Membrane binding rate of Scd1/2 (decreased from [4] to match decrease in r_S)
k_{ST}	0.015 /s	Membrane binding rate of Scd1/2 in cytoplasm to membrane bound Cdc42-GTP (decreased from [4])
ρ	0.05 μm	Bimolecular interaction radius [4]
σ	0.055 μm	Bimolecular unbinding distance (close to ρ, [4])
λ_{SD}	5.3 /s	Bimolecular rate for Scd1/2 + Cdc42-GDP on membrane (as for Bem1 complex [4])
λ_{ST}	9.6 /s	Bimolecular rate for Scd1/2 + Cdc42-GTP on membrane (as for Bem1 complex [4])
λ_{STT}	5 /s	Bimolecular rate for Scd1/2/Cdc42-GTP mediated activation of membrane bound Cdc42-GDP (lowered compared to [4])
λ_{sGAP}	10 /s	Bimolecular rate parameter for sGAP mediated hydrolysis of Cdc42-GTP (large enough to matter)
N_{Cdc42}	5000	Total number of Cdc42 particles (Estimates from total cell numbers in [5,6] and (PomBase.org), divided by 4 for sphere volume and considering a larger fraction of Cdc42 is bound to internal membranes)
$N_{Scd1/2}$	225	Total number of Scd1/2 particles (from Scd1 in [6] divided by 2, assumed to be tip biased)
N_{sGAP}	2250	Total number of sGAP particles (essentially arbitrary since λ_{sGAP} is tuned but comparable to sum of Rga4 = 646, and Rga6 = 1102 in [6])

Table S3. Model parameter values for membrane flows from [1] unless otherwise indicated.

A_{exo}	3.14 x 10^{-2} μm^2	Surface area of individual exocytotic vesicle
A_{endo}	6.42 x 10^{-3} μm^2	Surface area of individual endocytotic vesicle
r_{exo}	0.68 /s	Rate of exocytosis events per tip in growing *S. pombe*
r_{endo}	2.44 /s	Rate of endocytosis events per tip in growing *S. pombe*
w_{exo}	0.01 μm	Variance of blurring Gaussian for exocytosis (determined based on profiles from [2] and [1])
w_{endo}	1.51 μm	Variance of blurring Gaussian for endocytosis (determined based on profiles from [2] and [1])
α	0.5	Fraction of mobile component in membrane
γ	1.0	Coupling between the proteins and the flowing membrane
R_{cutoff}	2.0 μm	Cutoff for effect of exo/endocytosis

Table S4. Strain list

Strain number	Genotype	Reference
YSM3138	h+ leu1-32 cdc42-mCherry^{SW}:Term:kanMX ura4-294:pshk1:CRIB-3GFP:ura4+	[2]
YSM2468	h+ leu1-32 cdc42-mCherry^{SW}-1ritC:kanMX ura4-294:pshk1:CRIB-3GFP:ura4+	[2]
YSM4074	h+/h- ura4-/ura4+ ade6-M210/ade6-M216 cdc42-mCherry^{SW}-3ritC:Term:kanMX/cdc42+	This work
YSM4075	h+/h- ura4-/ura4+ ade6-M210/ade6-M216 cdc42-mCherry^{SW}-2ritC:Term:kanMX/cdc42+	This work
YSM4077	h+/h- ura4-D18 ade6-M210/ade6-M216 leu1-32:pshk1:CRIB-3GFP:ura4+:leu1+/leu1+ cdc42-mCherry^{SW}-3ritC:Term:kanMX/cdc42+	This work
YSM4078	h+/h- ura4-D18 ade6-M210/ade6-M216 leu1-32:pshk1:CRIB-3GFP:ura4+:leu1+/leu1+ cdc42-mCherry^{SW}-2ritC:Term:kanMX/cdc42+	This work
YSM4081	h+/h- ura4- ade6-M210/ade6-M216 leu1-32:pshk1:CRIB-3GFP:ura4+:leu1+/leu1- rga6Δ::bleMX/rga6+ rga4Δ::natMX/rga4+ rga3Δ::hphMX/rga3+ cdc42-mCherry^{SW}-3ritC:kanMX	This work
YSM4083	h+/h- ura4-D18 ade6-M210/ade6-M216 leu1-32/leu1-32:pshk1:CRIB-3GFP:ura4+:leu1+ cdc42-mCherry^{SW}-1ritC:kanMX/cdc42+	This work
YSM4084	ura4? ade6- cdc42-mCherry^{SW}-2ritC:Term:kanMX	This work
YSM4085	ura4-D18 ade6- leu1-32:pshk1:CRIB-3GFP:ura4+:leu1+ cdc42-mCherry^{SW}-2ritC:Term:kanMX	This work
YSM4087	ade6- rga6Δ::bleMX rga4Δ::natMX rga3Δ::hphMX cdc42-mCherry^{SW}-3ritC:kanMX leu1-32:pshk1:CRIB-3GFP:ura4+:leu1+	This work

SI References

1 Gerganova, V. *et al.* Cell patterning by secretion-induced plasma membrane flows. *Sci Adv* **7**, eabg6718 (2021). https://doi.org/10.1126/sciadv.abg6718

2 Bendezu, F. O. *et al.* Spontaneous Cdc42 polarization independent of GDI-mediated extraction and actin-based trafficking. *PLoS Biol* **13**, e1002097 (2015). https://doi.org/10.1371/journal.pbio.1002097

3 Bartnicki-Garcia, S., Garduno-Rosales, M., Delgado-Alvarez, D. L. & Mourino-Perez, R. R. Experimental measurement of endocytosis in fungal hyphae. *Fungal Genet Biol* **118**, 32-36 (2018). https://doi.org/10.1016/j.fgb.2018.07.001

4 Pablo, M., Ramirez, S. A. & Elston, T. C. Particle-based simulations of polarity establishment reveal stochastic promotion of Turing pattern formation. *PLoS Comput Biol* **14**, e1006016 (2018). https://doi.org/10.1371/journal.pcbi.1006016

5 Marguerat, S. *et al.* Quantitative analysis of fission yeast transcriptomes and proteomes in proliferating and quiescent cells. *Cell* **151**, 671-683 (2012). https://doi.org/10.1016/j.cell.2012.09.019

6 Carpy, A. *et al.* Absolute proteome and phosphoproteome dynamics during the cell cycle of Schizosaccharomyces pombe (Fission Yeast). *Mol Cell Proteomics* **13**, 1925-1936 (2014). https://doi.org/10.1074/mcp.M113.035824
